# Replicated Pattern Formation and Recognition Properties of 2,4-Dichlorophenoxyacetic Acid-Imprinted Polymers Using Colloidal Silica Array Molds

**DOI:** 10.3390/polym11081332

**Published:** 2019-08-11

**Authors:** Gita Amiria Aya, Jin Chul Yang, Suck Won Hong, Jin Young Park

**Affiliations:** 1 Polymer Science & Engineering, School of Applied Chemical Engineering, Kyungpook National University, 80 Daehak-ro, Buk-gu, Daegu 41566, Korea; 2 Department of Optics and Mechatronics Engineering, Department of Cogno-Mechatronics Engineering, Pusan National University, Busandaehak-ro 63 beon-gil 2, Geumjeong-gu, Busan 46241, Korea; 3 Department of Polymer Science & Engineering, Kyungpook National University, 80 Daehak-ro, Buk-gu, Daegu 41566, Korea

**Keywords:** molecular imprinting, photopolymerization, colloidal lithography, colloidal silica, herbicide

## Abstract

Surface imprinting is an effective and simple method to fabricate and retain imprinted templates and recognizable nanocavities after template extraction. The imprinted effects can be controlled depending on the surface morphological changes. In general, a planar film has a limited area compared to a structured film with relatively higher surface-to-volume (S/V) ratio (A/A_0_), leading to the conventional sensing response upon the functionality of monomers in a fixed chemical composition. To increase the limited sensing properties and develop simple fabrication of porous arrays on a large area, we herein demonstrate the 2,4-dichlorophenoxyacetic acid (2,4-D, herbicide)-imprinted porous thin film lithographically patterned using photopolymerization and silica colloidal array as a master mold, derived by a unidirectional rubbing method. The resonant frequency changes with respect to the adsorption of 2,4-D molecules on a template-extracted porous poly(MAA-co-EGDMA) (MIP) film in a 10^−1^ mM aqueous solution of 2,4-D for 1 h, and when compared to the planar MIP film, the higher sensing response (Δ*f* = −283 ± 7 Hz ≈ 1543 ± 38 ng/cm^2^) appears on the porous MIP film due to the specific recognition toward the more accessible templated cavities of the structured porous array, indicating an imprinting effect (I_f_) value of 3.5. In addition, a higher selectivity for 2,4-D was also displayed on the porous MIP film compared to other herbicides. From these results, it was revealed that these improved sensing properties can be determined from the effects of various parameters (template functionality, film structuring, hydroxyl groups of silica colloids, etc.).

## 1. Introduction

To date, various herbicides have been used extensively to increase agricultural production. However, the risks of large amounts of consumption threaten natural purification and ecosystems. Among the mostly used herbicides, in particular, 2,4–dichlorophenoxyacetic acid (2,4-D) is one of important phenoxy herbicides, commonly known and remarked as a priority pollutant [1]. This compound is moderately toxic and poorly biodegradable, and widely applicable to the general cereal crops such as grain, wheat, corn, and sorghum to remove broad-leaf weeds. A recent study suggests that long-term exposure to 2,4-D gives rise to serious health hazards for humans, leading to damages in multiple organ systems [1]. For these reasons, various methods of 2,4-D removal have been studied including electrochemical, advanced oxidation, and radiolytic decomposition processes. However, the adsorption approach has been mainly applied using various adsorbents, such as clay, activated carbon, polymer, silica gel, etc. [1]. There are many conventional techniques for detecting low concentrations of 2,4-D such as chromatography or mass spectrometry [2,3,4]. However, these techniques often require extensive sample preparations, high cost equipment, and separation procedures, which are time-consuming processes.

As a sensing platform, the molecular imprinting technique is a favorable route for easy access to a wide range of research areas such as electrochemical biosensors [5], biomimetic sensors [6], and drug extraction [7]. The general principle of MIPs is the formation of specific recognition sites (such as templated cavities) that are formed through the copolymerization process between the monomers and crosslinker in the presence of target molecules [8,9]. Complexes can be formed in a solution mixed together with a monomer and the target molecules, covalently or noncovalently linking via their functional groups. During the polymerization process, the crosslinking agent will arrange them to positions that produce highly crosslinked three-dimensional (3D) polymer networks. After the effective removal of templated molecules, particular 3D cavities are readily created depending on the shape, size, and arrangement of functional groups. To successfully achieve a continuous series of molecular imprinting processes, advantageous synthetic strategies have been developed to control the active binding sites of molecular imprinting such as photo- or electro-polymerization based on the noncovalent bonding approach. These approaches have been widely adopted to efficiently establish well-preserved binding sites in the MIP matrix, compared to bulk the MIP compound. Furthermore, all the steps in the process are straightforward, but efforts are still required to improve the efficiency and functionality of MIP systems. To improve the sensing properties, various parameters such as affinity, specificity, stability, and selectivity involved in the sensing systems (nanoparticles, beads, or thin films) have been deeply considered for further applications, such as biomedical engineering [10,11,12,13]. In MIP sensing systems, the chemical composition of functional monomers associated with template molecules is the most important factor required to design ideal noncovalent bonding. In general, more functional monomers (~4 fold compared to the template molecule in molar ratio) are highly desirable for establishing sufficient nanocavities to recognize specific templates, rather than theoretically calculated mass [14]. Recently, some efficient MIP-techniques based on surface imprinting have been extensively explored to utilize sufficient, stable nanocavities with higher accessibility unlike bulk imprinting approaches. The development of MIPs to monitor or remove herbicides (2,4-D from drinking water and human urine) has been accomplished using various forms of size-controlled nanomaterials such as quantum dots, nanoparticles, micro/nanostructures and nanofibers [15,16,17,18,19,20]. As part of surface imprinting, the design of surface structures or efficient patterning in the micro/nanoscale of MIP films can facilitate the improvement sensing signal levels due to increase in the surface-to-volume (S/V) ratio [21] and fast diffusion toward the surface of MIP films [22]. In this context, we have previously reported several efficient methods for increasing the effective surface area of MIP films using colloidal/soft lithography and photo-/electro-polymerization [23,24,25,26]. For example, the efficient utilization of highly ordered spherical colloidal arrays as sacrificial masks for MIP deposition enables the generation of well-defined and uniform nanostructures with a large S/V ratio. However, two-dimensional (2D) colloidal crystals are not easily formed over a large area.

Here, we report a robust strategy for the production of porous MIP thin films by fully utilizing nanostructured silica particle arrays as a mold that can be simply prepared by a unidirectional rubbing technique. Thus, well close-packed hexagonal arrays of the dry-type silica particles formed on a solid substrate were provided as a master template mold on a large scale [27,28]. This highly ordered crystal arrays can be extended to lithographic approaches geared toward the development of freestanding MIP films (or filter/membranes) for mass production using the roll-to-roll process. In particular, to fabricate patterned MIP films for the detection of 2,4-D, we used methacrylic acid (MAA) as functional monomer and ethylene glycol dimethacrylate (EGDMA) as crosslinker that were polymerized in the process of step-and-flash nanoimprint lithography under UV exposure. From gravimetric detection based on in situ quartz crystal microbalance (QCM), the sensing properties of nanopatterned MIP films were compared to planar MIP (*pl*-MIP) films as well as nonimprinted films. Furthermore, the apparent selectivity toward 2,4-D molecules compared to other analogous herbicides, including atrazine, ametryn, and glufosinate is investigated by evaluating the change in resonant frequency.

## 2. Materials and Methods

### 2.1. Materials

Tetraethyl orthosilicate (TEOS, 98%, analytical reagent) was purchased from Acros Organics. Absolute ethanol (99.9%, analytical reagent) and ammonia water (NH_4_OH, analytical reagent) were obtained from Sigma Aldrich Co., St. Louis, MO, USA. To prepare patterned MIP films, MAA (Daejung Chemicals & Metals Co., Siheung, Korea) was used as a functional monomer. EGDMA, 2,4-dichlorophenoxyacetic acid (2,4-D; M_w_ 221.04 g/mol), and dimethylformamide (DMF) were purchased from Tokyo Chemical Industry Co (Tokyo, Japan). use as cross-linker, template molecule, and solvent, respectively. 2,2′-azobisisobutyronitrile (AIBN), used as an initiator, was purchased from Daejung Chemicals & Metals Co. Atrazine, ametryn, glufosinate (Sigma-Aldrich Co.), and (4-chloro-2-methylphenoxy)acetic acid (MCPA, Tokyo Chemical Industry Co.) were used as 2,4-D-analogous chemicals for the MIPs’ selectivity test. Deionized (DI) water (Pure & Ultra pure water system, pure ROUP30, Pure Water Co., Namyangju, Korea) and all other chemicals were used as received.

### 2.2. Synthesis of Monodispersed Silica Particles

The monodispersed silica particles were prepared using various methods, including the reverse microemulsion method and chemical vapor condensation [29,30,31]. For colloidal particles, the well-known Stöber method was used to synthesize uniform-sized silica particles due to its simplicity and ease of preparation [32]. The synthesis of silica particles was performed as follows. First, 11 mL of TEOS dissolved in 200 mL of absolute ethanol was stirred for 10 min, and 120 mL of NH_4_OH was added into this mixture. The reaction temperature was then increased to 100 °C under magnetic stirring for 4 h to generate a uniform silica particle size. The obtained silica particles were centrifugally separated from suspension, washed with absolute ethanol several times, and placed overnight in the oven at 50 °C.

### 2.3. Fabrication of p-MIP Films

To prepare a polydimethylsiloxane (PDMS) freestanding substrate, a 10:1 weight ratio of silicone elastomer and curing agent (Sylgard 184, Dow Corning Co., Midland, MI, USA) was firmly mixed, and then the entrapped air bubbles were discarded via vacuum degassing. It was carefully poured into a plastic petri dish and placed in the oven at 60 °C for 2–3 h. After thermal curing, the PDMS was left to cool at room temperature and then cut to a small cubic size (15 × 15 mm^2^). A closed-packing colloidal array of silica monolayer was then prepared on one of the two PDMS substrates via a unidirectional rubbing method [28]. Initially, dry powder of the synthesized silica particles was spread on top of one PDMS substrate and wiped with the other PDMS substrate at a randomly selected direction. Shortly after being wiped, hexagonally ordered silica arrays were assembled on the surface of both PDMS substrates.

Structured MIP film formation on 9 MHz AT-cut quartz crystal (Seiko EG&G, Seiko Instruments Inc., Chiba, Japan) was performed via in situ photopolymerization to generate an imprinted polymer matrix from monomer precursors confined within a small reaction volume under a UV light. The precursor solution was prepared by adding AIBN (1 mM) in 50 µL of DMF into MAA (4 mM), 2,4-D (1 mM), and EDMA (20 mM) in the 20 mL vial. Then, it was degassed under nitrogen gas for 10–15 min. The precuring step under a UV light (370 nm, 36 W) for 70 s was performed to generate a slightly viscous solution for the next step. First, 1.5 µl of the precured solution was dropped onto the silica colloidal arrays assembled on the PDMS substrate, and a clean quartz substrate was carefully brought into contact with the PDMS substrate. Sufficient pressure was applied on top of the quartz substrate to obtain the complete contact between two substrates for the formation of a better MIP replica film, then, a UV light was immediately induced for 7 min. After complete polymerization, the quartz substrate was peeled off and dried at room temperature. The obtained porous MIP (*p*-MIP) film was immersed into a 4 wt% hydrofluoric acid solution for 30 min to diminish the silica particles, and was rinsed with distilled water several times [11]. Subsequently, the extraction of 2,4-D was performed by soaking the *p*-MIP film into the methanol for another 2 h to breach the presence of hydrogen bonding between the carboxylic group of MAA and carboxylic group of 2,4-D. After 2 h, it was continuously washed with methanol several times. In the same way, the nonimprinted *p*-MIP film was fabricated correspondingly with the imprinted *p*-MIP film without the presence of a target molecule.

### 2.4. Characteristics

The size distribution of the silica colloidal particles and surface topographies of *p*-MIP/NIP films were characterized using field emission scanning electron microscope (FE-SEM, Hitachi SU8220, Tokyo, Japan) and atomic force microscope (AFM, NX20, Park Systems, Suwon, Korea). To examine the sensitivity and selectivity of 2,4-D, the change in resonant frequency was monitored via a QCA 922 quartz crystal analyzer for 1 h.

## 3. Results and Discussion

**Figure 1:**
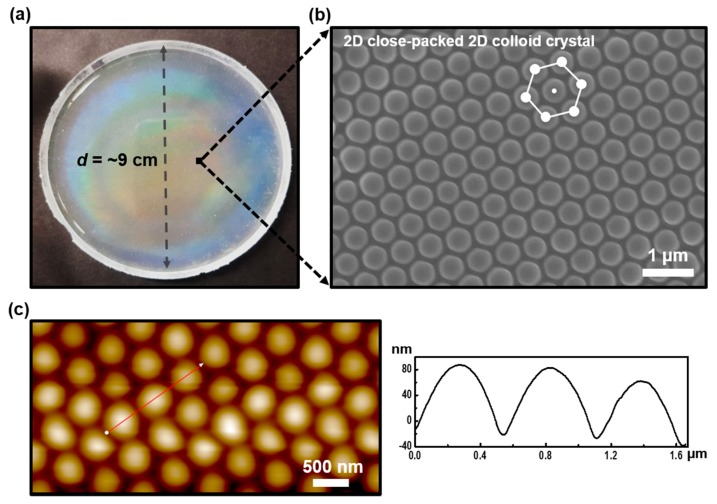
(**a**) Digital image of the monolayer 2D silica colloidal array film on a large scale, fabricated on the PDMS substrate by the directional rubbing method. (**b**) SEM and (**c**) AFM images of well-organized silica colloidal arrays with hexagonally ordered structures, and the height profile is presented besides the AFM image. The scale bar in (**b**) is 1 μm.

In order to prepare monodispersed silica colloidal particles having a uniform size distribution, a few parameters were precisely controlled, including reaction temperature and concentration of TEOS, ethanol, and ammonia according to the Stöber method [32]. The size-defined silica particles were produced with an average diameter of 490 ± 14 nm under the optimized synthetic condition (Figure S1). Using these colloids, the unidirectional rubbing method was utilized to generate a 2D silica colloidal film on a PDMS substrate [28]. In the rubbing process along the selected direction, initial colloidal grains were assembled into a monolayer, and the diffraction of light, a typical feature of photonic crystals, appeared due to the closely packed silica colloidal geometry (Figure 1a). As shown in Figure 1b, the representative image measured by electron scanning microscopy (SEM) confirmed that the silica particles were formed with a 2D hexagonal structure over a large area on the PDMS substrate. To clarify the dimensional features of the nanostructured surface, AFM measurements were performed. A relatively uniform well-ordered array of silica grains was revealed in the size range of 480–500 nm as shown in Figure 1c. As a result, the 2D silica colloidal film used as a master mold, designed for nanoimprint lithography of MIPs, was successfully fabricated by the directional rubbing process.

**Figure 2:**
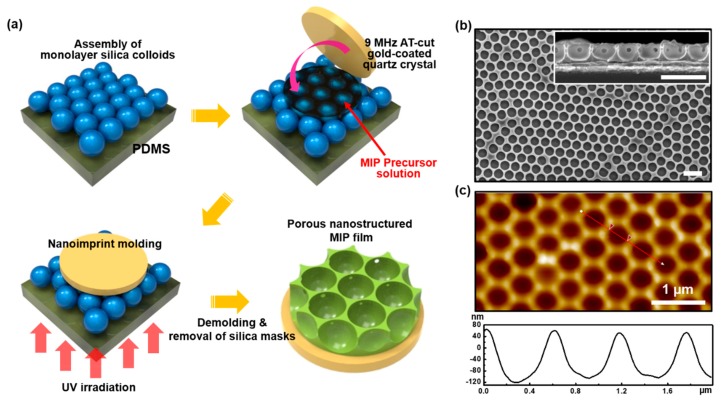
(**a**) Fabrication process of a porous MIP (*p*-MIP) film using a 2D silica colloidal monolayer as a master mold, (**b**) SEM (inset is a cross-sectional image), and (**c**) AFM images (line profilometry is included below the AFM image) of the *p*-MIP film. All the scale bars in (**b**) are 1 μm.

Using the silica colloidal array as a master mold, replicated nanostructured MIP films were manipulated as schematically illustrated in Figure 2a. After dropping a certain amount of MIP precursor solution on the mold, a gold-coated quartz crystal was conformally placed on top of the mold. Subsequently, UV irradiation was applied through the bottom PDMS surface for the photopolymerization. After demolding the quartz crystal and removal of silica colloids, the nanostructured MIP film was successfully produced. As shown in Figure 2b, hexagonally ordered porous arrays of MIP (*p*-MIP) were observed, and the size of pores was defined as ~450 nm and ~350 nm in diameter and depth, respectively. The small holes in the cross-sectional SEM image in the inset of Figure 2b are formed from the tightly contacted area between the two silica particles in the process of eliminating colloids. When the corresponding surface was measured by AFM, the almost identical porous structures appeared (Figure 2c). The depth of the pores was at a slightly lower level (~180 nm) probably due to the AFM tip effect during the scanning process [33]. A nonimprinted polymer (NIP) films were also prepared using the same precursor solution without 2,4-D molecules to compare the sensing results with the *p*-MIP film. The surface morphology and geometrical dimension of *p*-NIP film were exactly identical to those of the *p*-MIP films (see Figure S2). Moreover, both *pl*-MIP and *pl*-NIP films were prepared as control samples with a thickness of ~85 nm using the same precursor solutions (Figure S3).

**Figure 3:**
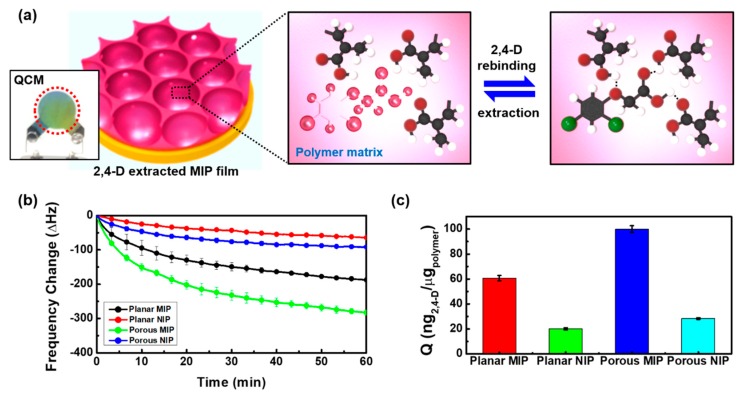
(**a**) Scheme of the rebinding and extraction process on the 2,4-D-extracted MIP film, (**b**) frequency change as a function of time on the *p*-MIP/NIP and *pl*-MIP/NIP films in 10^−1^ mM 2,4-D aqueous solution for the 1 h rebinding process (n = 3), (**c**) Q values (adsorbed 2,4-D mass (ng) per poly(MAA-co-EGDMA) unit weight (μg)) for the four samples.

In our 2,4-D imprinted MIP system, the functional MAA monomer works as a robust H-bond acceptor and can be linked to the carboxylic group of 2,4-D molecules via noncovalent interactions (hydrogen bonding) in a polymer network. Immobilization of the MAA monomer and 2,4-D molecules is for the purpose of forming complexes. Therefore, the extraction of 2,4-D allows MIP films to create cavities or interactions with the recognizable template (Figure 3a). On the basis of the noncovalent interactions, the sensing behaviors of 2,4-D imprinted *p*-MIP and *p*-NIP films on gold-coated quartz crystals were examined by measuring the resonant frequency changes of QCM signals in an aqueous solution of 10^−1^ mM 2,4-D. The *pl*-MIP and *pl*-NIP films were also investigated in the same solution as controls.

Once the 9 MHz AT-cut gold-coated quartz crystals is exposed to air, its sensitivity factor becomes approximately 0.1834 Hz/(ng/cm^2^) [34]. Based on the defined gold area (5 mm-diameter, A = 0.19625 cm^2^), the 1 Hz decrease in frequency shift is almost equal to 1.07 ng in mass loading. From the frequency change between the bare and mass-loaded (film deposition) quartz crystal (Δ*f_p_*_-MIP_ = −3330 ± 4 Hz, Δ*f_pl_*_-MIP_ = −3487 ± 13 Hz, Δ*f_p_*_-NIP_ = −3477 ± 15 Hz, and Δ*f_pl_*_-NIP_ = −3432 ± 1 Hz) measured in air, it was revealed that the films with 3.6–3.7 μg mass were loaded on the defined gold area on each quartz crystal. After the removal of imprinted 2,4-D molecules by soaking them in methanol for 2 h, resonant frequency in all the MIP films (Δ*f_p_*_-MIP-ex_ = 302 ± 8 Hz and Δ*f_pl_*_-MIP-ex_ = 184 ± 7 Hz) increased due to their diffusion into the solution. From this extracted template mass, validated binding capacity was calculated as 323 ± 9 ng and 197 ± 7 ng for the *p*- and *pl*-MIP films, respectively. However, the frequency change of both NIP films was negligibly smaller (Δ*f*_NIP_ = 12 Hz, corresponding to 13 ng). Moreover, the slight increase in frequency, even though there are no 2,4-D molecules in the NIP films, may have originated from the elimination of unreacted monomers under UV irradiation during photopolymerization.

Figure 3b represents the frequency changes with respect to the adsorption of 2,4-D molecules on template-extracted *pl*-MIP and *p*-MIP films in a 10^−1^ mM aqueous solution of 2,4-D for 1 h. Since the molecular-imprinted films are considered rigid film due to the excessive use of crosslinker, resonant frequency changed according to Sauerbrey’s equation [35]. Commonly, when one side of a quartz crystal is in contact with a liquid, frequency change depends on its density and viscosity. Therefore, total frequency shift (Δ*f* = Δ*f_m_* + Δ*f_l_*) can be determined by the combined mass and liquid loading effect [36]. However, mass loading on the rigid film by 2,4-D adsorption is primarily reflected in the frequency change from initial constant frequency (*f*_0,*l*_) lowered after being in contact with an aqueous solution.

As a control sample, the *pl*-MIP film showed a frequency change of −187 ± 7 Hz, corresponding to the mass per unit area (ng/cm^2^) of 1020 ± 38. However, the higher sensing response (Δ*f* = −283 ± 7 Hz ≈ 1543 ± 38 ng/cm^2^) appeared on the *p*-MIP films due to the specific recognition toward the more accessible templated cavities of the nanostructured porous arrays. In other words, this could be explained by an increase in the effective surface area as a result of the nanoporous structuring process. It was comparatively favorable for the rapid transport of 2,4-D target molecules in imprinted film and allowed the specific recognition of 2,4-D over the nanocavities distributed over the entire thin film’s surface. In addition, the hydroxyl groups on the surface of the silica colloids are involved in complex formation during the polymerization process. For NIP films, nonspecific template adsorption on the surface occurred due to the unrecognizable cavities in the polymer matrix, representing significantly lower sensing responses (Δ*f* = −64 ± 3 Hz and −92 ± 2 Hz) on the *pl*-NIP and *p*-NIP films. When the formation process of MIP and NIP films is considered, the conformation of the polymer matrix in the formed MIP film may be affected by existing interactions with the template, and template recognition in the cavities preferentially occurs in the rebinding process due to the existing affinity and template specificity. However, nonspecific binding on the surface of MIP films, as a minor effect, also influences the frequency change in the rebinding process. As shown in Figure S4, using a nonextracted *pl*-MIP film, we explored a rebinding process on the same 2,4-D solution to investigate nonspecific adsorption. Even though the imprinted templates were fully occupied in the cavities, 2,4-D molecules were nonspecifically adsorbed on the surface, indicating that the lower frequency change of −28 ± 8 Hz appears in comparison to that of the *pl*-NIP film. Therefore, in the case of *p*-MIP films, the conformation of imprinted films can be determined from the effects of the template’s functionality, thin film structuring, and the hydroxyl groups of silica colloids, consequentially amplifying the sensing signal and indicating enhanced sensing properties.

As shown in Figure 3c, the sensing responses were converted to Q values (mass of templated molecules to the MAA-co-EGDMA polymer), corresponding to 57 ± 2 ng/μg for *pl*-MIP and 93 ± 2 ng/μg for *p*-MIP. Even though the MIP films were formed with the same monomer composition, the major effect of hydroxyl groups on the silica colloids as well as the minor effect of increased effective surface area could reflect in the molecular conformation and cavities formation, associated with non-covalent bonding between functional groups of templated polymer and templates. Interestingly, the Q values of *p*-MIP were greatly improved compared to those of MIP micromonoliths (0.045 ± 0.015 μmol/g) prepared by micromolding in capillaries (MIMIC) using a PDMS stamp in the reported literature [18]. The MIMIC process allows only a microscale for MIP patterns because the MIP precursor must be able to flow into the mold by capillary force. Moreover, the MIP film is not formed on the contact surface between the PDMS and the substrate. However, *p*-MIP has enhanced the sensing property for 2,4-D recognition owing to the high surface area and the effect of hydroxyl groups on the silica colloids without the limitations of MIP micromonoliths. In addition, Q values (Q*_pl_*_-NIP_ = 18.8 ng/ug and Q*_p_*_-NIP_ = 26.5 ng/ug) of the two NIP films were significantly lower than those of the MIP films. However, considering nonspecific adsorption behaviors on the NIP films, the two Q values are not identical in spite of the synthesized nonimprinted films having the same composition. Difference in Q values may be affected by increased effective surface area in the porous structure. From the surface area measured by AFM (Figure 2c and Figure S5), the surface-to-volume (S/V) ratio of patterned area to planar area (A/A_0_ = 0.26839/0.19625 cm^2^) was calculated to be ~1.37. Therefore, when applying the increased surface ratio to the Q value of the *p*-NIP film, the value obtained (25.8 ng/μg) is extremely close to the Q value of the *p*-NIP film, indicating that the molecules can diffuse onto the NIP films and that the nonspecific adsorbed mass increases in proportion to the surface area. These results show that 2,4-D molecules may not penetrate the highly crosslinked polymer matrix, and that nonspecific adsorption occurs only on the surface of the NIP films, leading to proportional increase in the sensing response with the increase in surface area. This effect also influences the imprinted factor (I_f_); Q_MIP_/Q_NIP_ [37]. In the *pl*-MIP film, the value of I_f_ is close to 3.0. However, in spite the increase in surface area, the porous MIP film had an I_f_ value of 3.4. A higher imprinting factor implies that the imprinted film can more sufficiently retain the target molecules compared to the nonimprinted film. On the other hand, if the MIP film’s I_f_ had a value close to 1 or equal to 1, this indicates that the MIP film’s capability of adsorbing the template molecule is similar to that of the NIP film. Recovery values on the *p*-MIP and *pl*-MIP films were 93.81% and 102.03%, with corresponding relative standard deviation (RSD%, n = 3) of 2.48 and 3.55, respectively. However, these recovery values include a minority of nonspecific adsorption mass on the surface. Thus, the values indicate that when making allowances for unsaturated frequency during the 1 h measurements, recognizable empty cavities can still exist in the film, and template molecules are not as fully detected as the maximum capacity of the MIP film.

**Figure 4:**
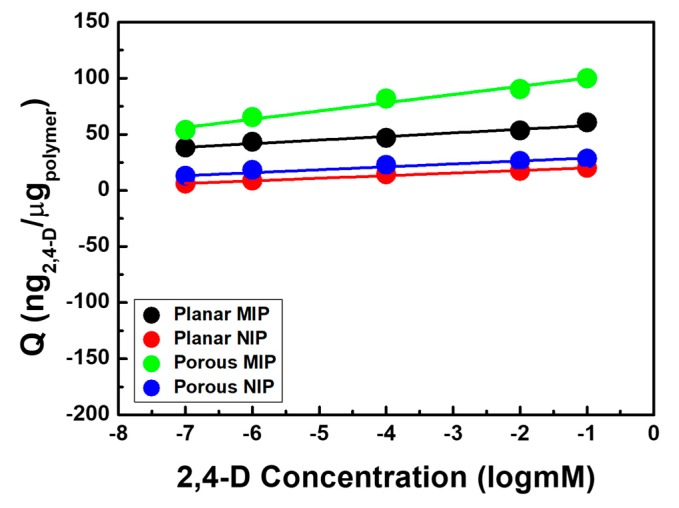
Q values (adsorbed 2,4-D mass (ng) per poly(MAA-co-EGDMA) unit weight (μg)) on the *p*-MIP/NIP and *pl*-MIP/NIP films as a logarithm function of 2,4-D’s concentration, and the concentration of 2,4-D ranges from 10^−7^ to 10^−1^ mM (*n* = 3).

To explore the sensitivity of 2,4-D detectable MIP films, resonant frequency changes were monitored in 2,4-D aqueous solutions with a concentration range of 10^−7^–10^−1^ mM (Figure S6 and Figure 4). Both MIP films showed increase in frequency changes with the increase in 2,4-D’s concentration. For more clarity, the *y*-axis was converted into Q values and the calibration curves were linear with a coefficient of determinations (R^2^) of 0.969 (*p*-MIP) and 0.958 (*pl*-MIP). Moreover, the sensitivities obtained were ~7.34 and 3.2 ng/(μg·log(mM)), respectively. The imprinted polymers resulted in higher binding amounts compared to the nonimprinted polymers due to the imprinting effect, which created cavities that precisely fit 2,4-D molecules with regard to the spatial structure and functional groups during the polymerization process.

**Figure 5:**
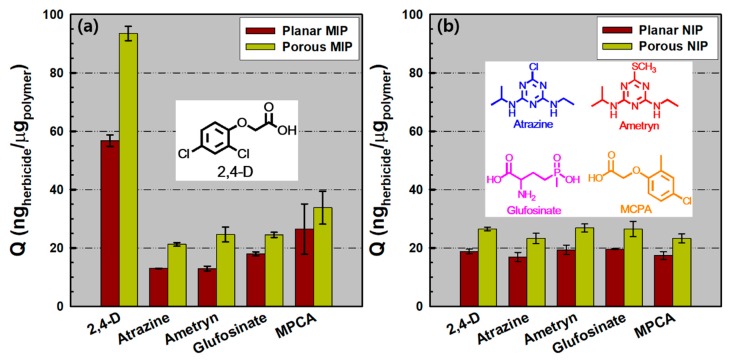
Q values as a function of time on (**a**) the *pl*- and *p*-MIP films and (**b**) *pl*- and *p*-NIP films in each analogous herbicide solution (2,4-D, atrazine, ametryn, glufosinate, or MCPA) with a fixed concentration (10^−1^ mM) for the 1 h rebinding process (*n* = 3).

Specific selectivity could be the main characteristic parameter for verifying the efficiency of the 2,4-D imprinted sensor. Using the *p*-MIP/NIP and *pl*-MIP/NIP films, frequency changes were explored in a 10^−1^ mM analogous herbicide solutions such as atrazine, ametryn, glufosinate, MCPA, and 2,4-D for 1 h (Figure S7). From the Q values described in Figure 5a, we found that the sensing responses of the four similar herbicides on the *p*-MIP film ranged from 21 to 34 ng/μg, derived from the similarity in chemical structures. However, in the case of *pl*-MIP films, relatively lower sensing responses appeared (13–26 ng/μg) due to nonspecific binding toward the limited area of the planar film. In the case of the glufosinate molecules, the selectivity effect values (S*_e_* = Q_MIP,2,4-D_/Q_MIP,glufosinate_) of *pl*-MIP and *p*-MIP films were ~3.16 and 3.82, respectively. However, on the basis of the MPCA molecules, with the most similar structure (chlorophenoxyacetic acid) to 2,4-D, the selectivity effect values (S*_e_* = Q_MIP,2,4-D_/Q_MIP,MPCA_) of *pl*-MIP and *p*-MIP films were ~2.15 and 2.77, respectively. These data show that the porous-structured MIP films result in enhanced selectivity over the *pl*-MIP film, and the S*_e_* values on both MIP films were highly dependent on the structural similarity and functionality of the analogous molecules. By comparison, when the NIP films were applied under the same solution conditions, the two NIP films exhibited Q values less than 27 ng/μg (regardless of herbicides) due to nonspecific binding only on planar or porous film surfaces (Figure 5b). This indicates that selectivity is remarkably reduced with NIP films. This result suggests that the sensing properties of MIP films can be significantly improved by a pattern formation strategy in nanoscale, as presented here.

## 4. Conclusions

In summary, we developed a facile method to fabricate porous nanostructured MIP films by fully utilizing hexagonally closed-packed silica spherical particle arrays as a mold. These highly ordered arrays of clear field mask were applied to lithographic approaches with the aim of developing porous MIP films for the detection of 2,4-D—the prototype of a large scale *p*-MIP film on the PDMS mold as described in Figure S8. The characteristic sensing properties of the response by exposing the target molecule 2,4-D were evaluated using the QCM-based gravimetric method. Compared to the *pl*-MIP film, these porous nanostructured MIP films clearly show improved sensing properties, such as amplified frequency signal, sensitivity, and selectivity, due to the easily accessible cavities that are related to proper conformation. Furthermore, other NIP films were also investigated, which revealed that nonspecific binding occurred only on the film surface. Conclusively, our extremely simple and cost effective strategy for producing a specific nanoimprint mold and successive photopolymerization of MIP films enables possible applications by templating functional multimolecules in a polymer matrix for a multidetectable sensing platform in the near future.

## Supplementary Materials

The following are available online at https://www.mdpi.com/2073-4360/11/8/1332/s1. Figure S1. SEM image of silica colloids; Figure S2. AFM and SEM images of *p*-NIP; Figure S3. Cross-sectional SEM images of the *pl*-MIP and *pl*-NIP films; Figure S4. Frequency change as a function of time on the *p*-MIP film with 2,4-D templates; Figure S5. Surface analysis of the *p*-MIP film; Figure S6. Frequency change as a function of time on the *pl*-MIP/NIP and *p*-MIP/NIP films in a variety of 2,4-D concentrations; Figure S7. Frequency change as a function of time on the *pl*-MIP/NIP and *p*-MIP/NIP films in each analogous herbicide solution; Figure S8. Photograph of the large scale *p*-MIP film on a polyethylene terephthalate (PET)-supporting film.
