# Supplementary material for: Replicated Pattern Formation and Recognition Properties of 2,4-Dichlorophenoxyacetic Acid-Imprinted Polymers Using Colloidal Silica Array Molds
Source: Polymers (Basel). 2019 Aug 11;11(8):1332. doi: 10.3390/polym11081332 (PMC6722725; doi:10.3390/polym11081332)
Supplement: Supplementary file 1 [file polymers-11-01332-s001.pdf]

Supporting Information

Replicated Pattern Formation and Recognition Properties of 2,4-Dichlorophenoxyacetic acid-Imprinted Polymers using Colloidal Silica Array Molds

Gita Amiria Aya [1], Jin Chul Yang [1], Suck Won Hong [2,*] and Jin Young Park [1,3,*]

1 Major in Polymer Science & Engineering, School of Applied Chemical Engineering, Kyungpook National University, 80 Daehak-ro, Buk-gu, Daegu 41566, Republic of Korea; gitaamiria@naver.com (G.A.A.), whdns861223@knu.ac.kr (J.C.Y.)

2 Department of Optics and Mechatronics Engineering, Department of Cogno-Mechatronics Engineering, Pusan National University, Busandaehak-ro 63 beon-gil 2, Geumjeong-gu, Busan 46241, Republic of Korea; swhong@pusan.ac.kr

3 Department of Polymer Science & Engineering, Kyungpook National University, 80 Daehak-ro, Buk-gu, Daegu 41566, Republic of Korea; jinpark@knu.ac.kr

* Correspondence: swhong@pusan.ac.kr; Tel.: +82-51-510-6119 (S.W.H.), jinpark@knu.ac.kr; Tel.: +82-53-950-5624 (J.Y.P.)

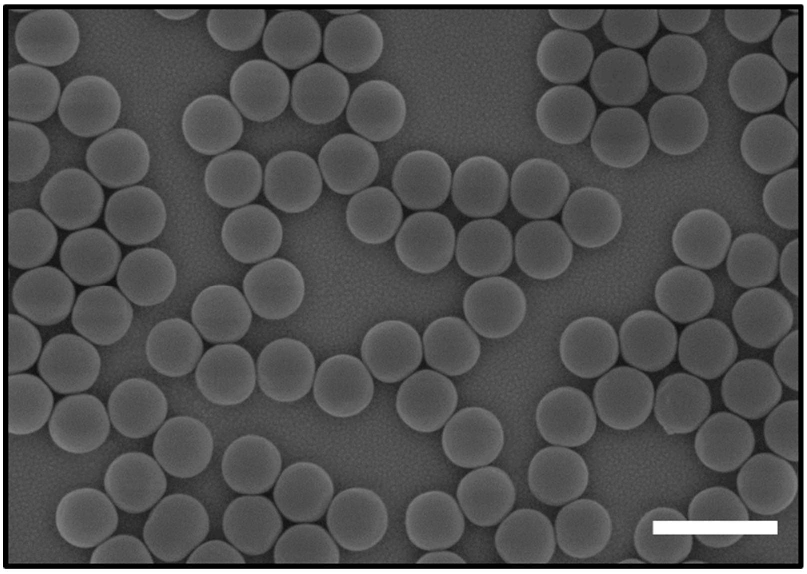

Figure S1. SEM image of synthesized silica colloids (the scale bar is 1 μm).

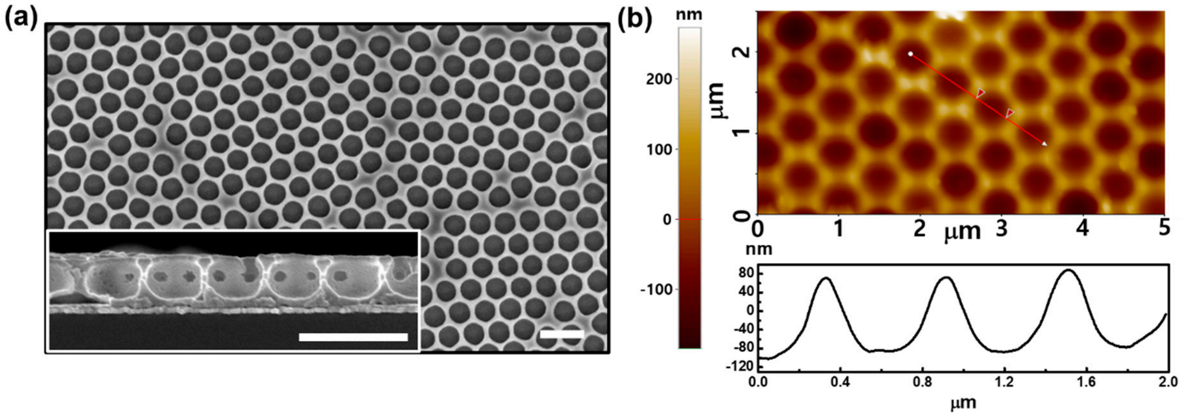

Figure S2. (**a**) SEM (inset is a cross-sectional image) and (**b**) AFM images (line profilometry is included below AFM image) of the *p*-NIP film. All the scale bars in Fig. S2 are 1 μm.

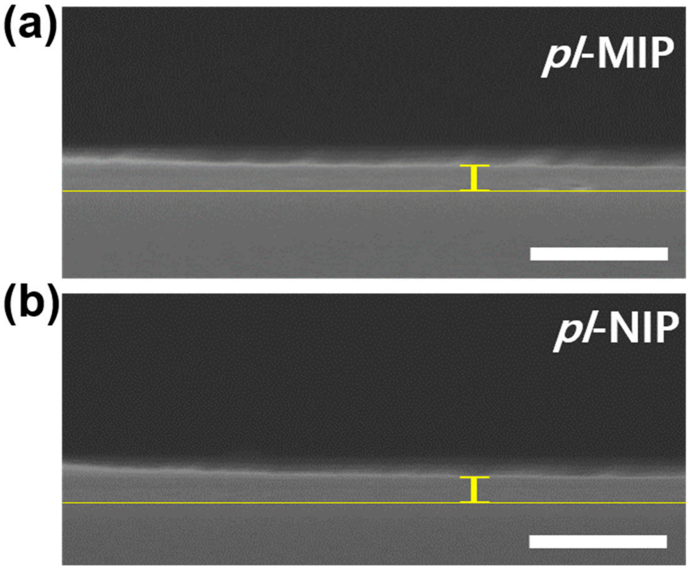

Figure S3. Cross-sectional SEM images of (**a**) *pl*-MIP and (**b**) *pl*-NIP films (all the scale bars in Fig. S3 are 500 nm).

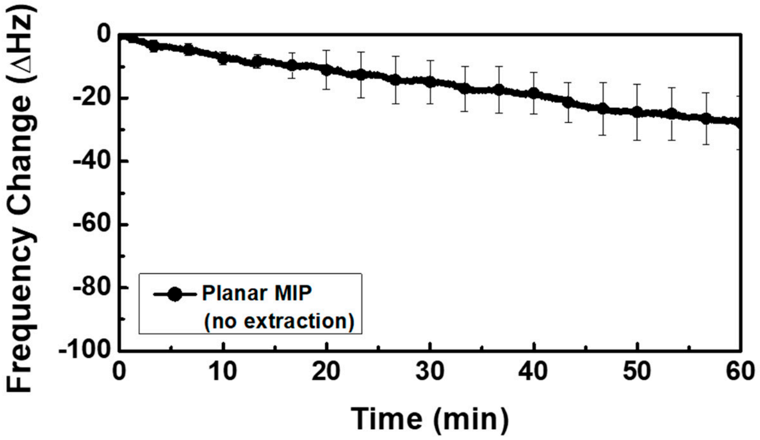

Figure S4. Frequency change as a function of time on the *p*-MIP film not undergoing the process of template extraction during rebinding experiment in 10^{-1} mM 2,4-D aqueous solution for 1 h.

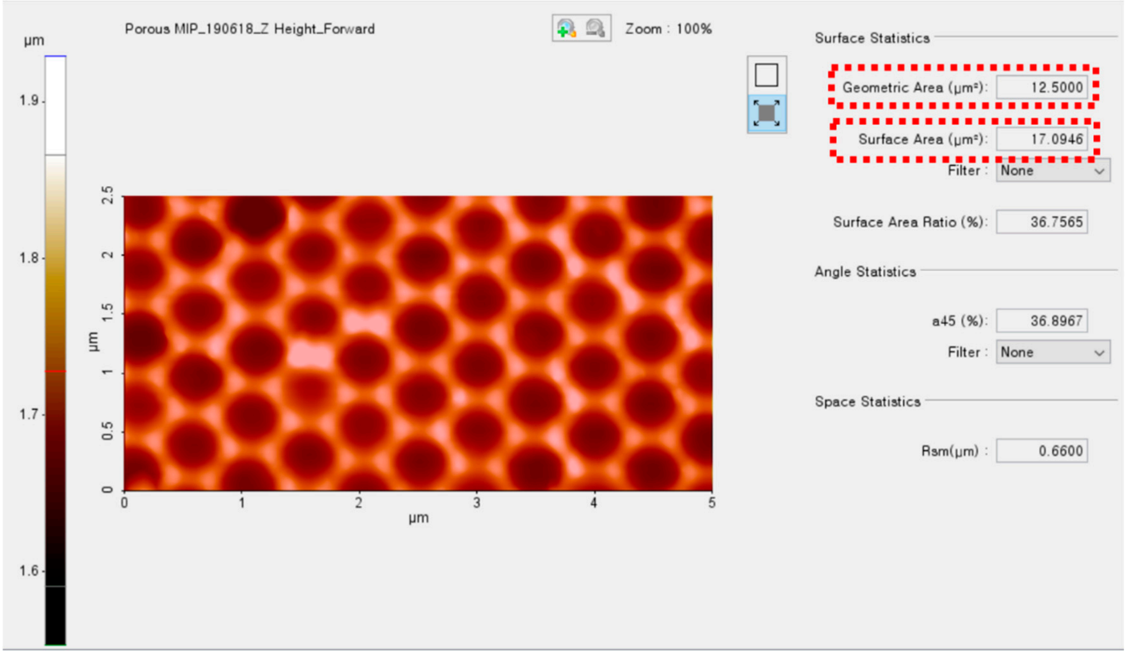

Figure S5. Surface analysis of the *p*-MIP film (note (red dash rectangle): the patterned surface area (17.0946 μm^2) on the scanned area (12.5 μm^2) indicates the increased aspect ratio of 1.37 (A/A_0) on the defined area.

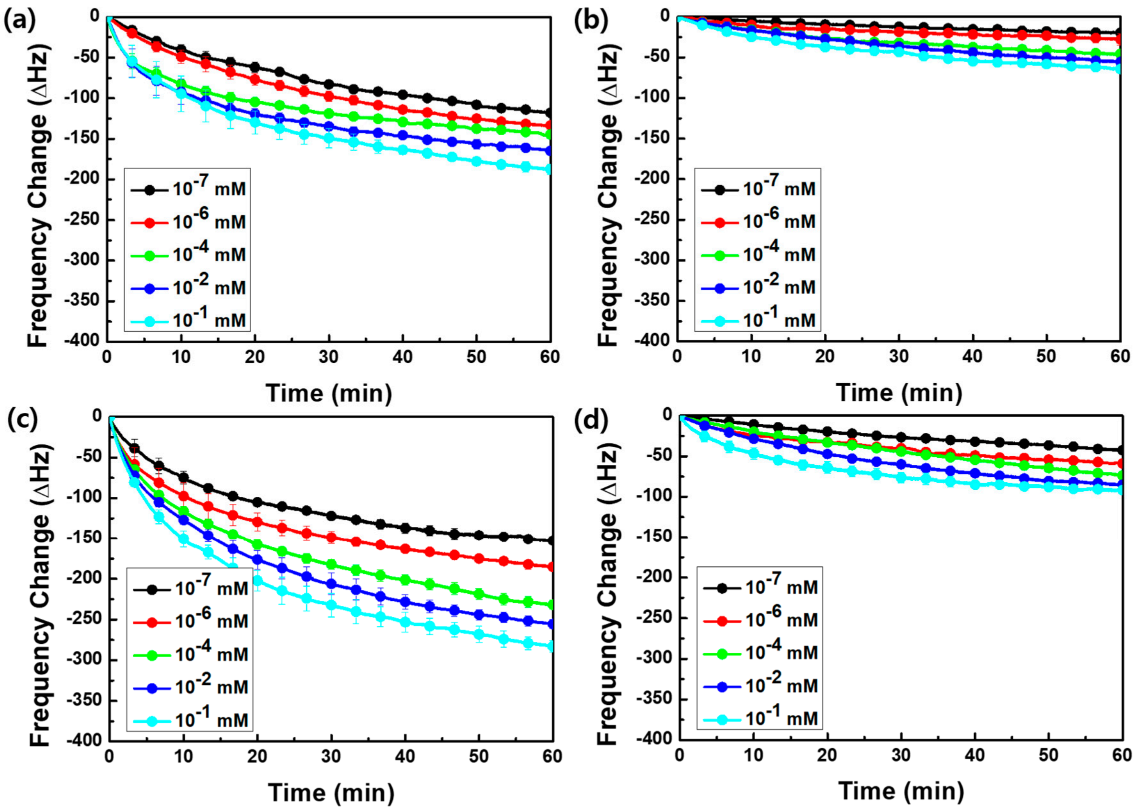

Figure S6. Frequency change as a function of time on (**a**) the *pl*-MIP, (**b**) *pl*-NIP, (**c**) *p*-MIP, and (**d**) *p*-NIP films in a variety of 2,4-D concentrations (10^{-7} – 10^{-1} mM) for the 1 h rebinding process.

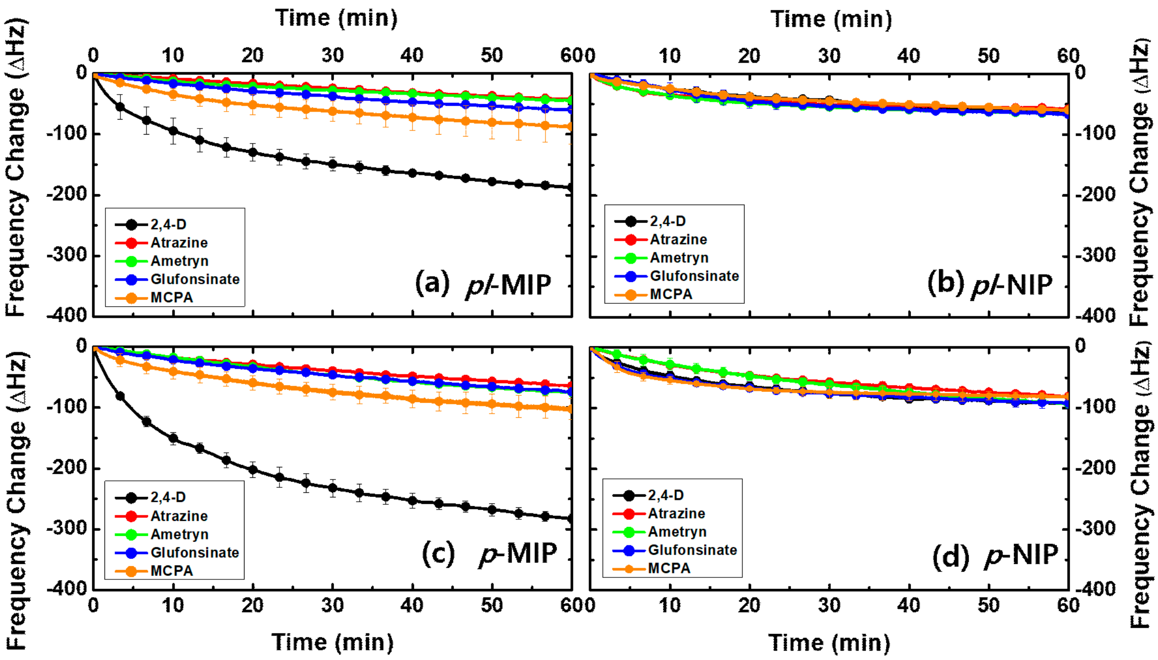

Figure S7. Frequency change as a function of time on (**a**) the *pl*-MIP, (**b**) *pl*-NIP, (**c**) *p*-MIP, and (**d**) *p*-NIP films in each analogous herbicide solution (2,4-D, atrazine, ametryn, glufosinate, or MCPA) with a fixed concentration (10^{-1} mM) for the 1 h rebinding process.

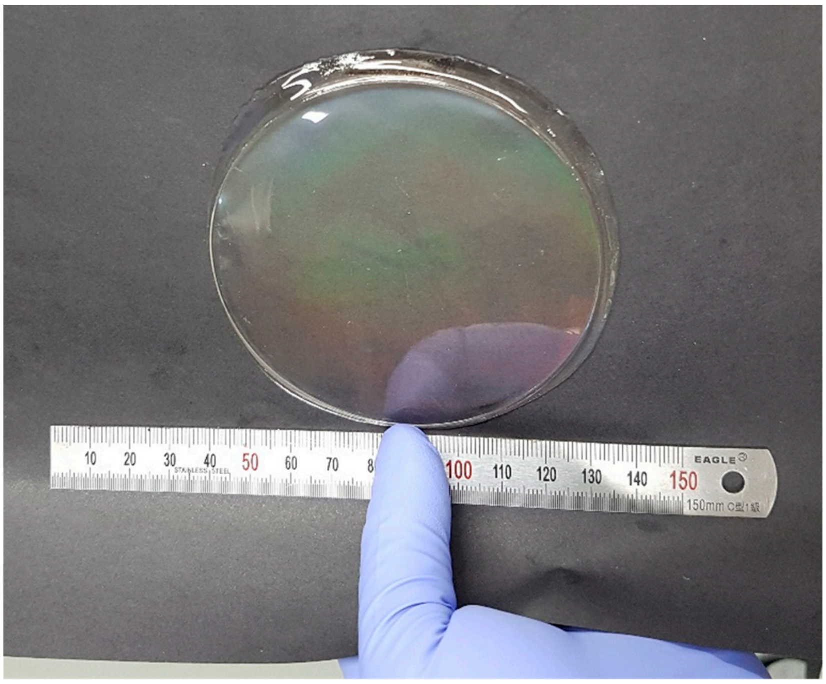

Figure S8. Photograph of the large scale (*d* = 8.9 cm) *p*-MIP film on a PET supporting film (a mixture solution (0.1 mmol 2,4-D, 0.4 mmol MAA, 1 mmol diurethane dimethacrylate, 1 mmol trimethylolpropane propoxylate triacrylate (TMPPTA), 0.1 mmol 1-hydroxycyclohexyl phenyl ketone (HCPK)) is used and UV irradiation is induced for 7 min).
